# Generative adversarial networks with adaptive normalization for synthesizing T2-weighted magnetic resonance images from diffusion-weighted images

**DOI:** 10.3389/fnins.2022.1058487

**Published:** 2022-11-14

**Authors:** Yanyan Mao, Chao Chen, Zhenjie Wang, Dapeng Cheng, Panlu You, Xingdan Huang, Baosheng Zhang, Feng Zhao

**Affiliations:** ^1^College of Oceanography and Space Informatics, China University of Petroleum, Qingdao, China; ^2^School of Computer Science and Technology, Shandong Business and Technology University, Yantai, China; ^3^Shandong Co-Innovation Center of Future Intelligent Computing, Yantai, China; ^4^School of Statistics, Shandong Business and Technology University, Yantai, China

**Keywords:** magnetic resonance imaging (MRI), images synthesis, generative adversarial network (GAN), image fusion, adaptive normalization

## Abstract

Recently, attention has been drawn toward brain imaging technology in the medical field, among which MRI plays a vital role in clinical diagnosis and lesion analysis of brain diseases. Different sequences of MR images provide more comprehensive information and help doctors to make accurate clinical diagnoses. However, their costs are particularly high. For many image-to-image synthesis methods in the medical field, supervised learning-based methods require labeled datasets, which are often difficult to obtain. Therefore, we propose an unsupervised learning-based generative adversarial network with adaptive normalization (AN-GAN) for synthesizing T2-weighted MR images from rapidly scanned diffusion-weighted imaging (DWI) MR images. In contrast to the existing methods, deep semantic information is extracted from the high-frequency information of original sequence images, which are then added to the feature map in deconvolution layers as a modality mask vector. This image fusion operation results in better feature maps and guides the training of GANs. Furthermore, to better preserve semantic information against common normalization layers, we introduce AN, a conditional normalization layer that modulates the activations using the fused feature map. Experimental results show that our method of synthesizing T2 images has a better perceptual quality and better detail than the other state-of-the-art methods.

## Introduction

Magnetic resonance imaging (MRI) has been used worldwide for the diagnosis of various conditions throughout the body, among which the brain and spine are the most effective. The magnetic field strength of MRI scanners has evolved from less than 0.5 T in the 1980s to the extensively used 1.5 and 3 T, and even 7 T (Van der Kolk et al., 2013; Lian et al., 2018). Different sequences of MR images from MRI scanners help doctors make more accurate decision. While doctors desire different sequences of images, certain conditions (e.g., medical conditions, patients’ physical conditions, costs) result in sacrificing some imaging sequences to complete the scan (Shen et al., 2017). For example, diffusion-weighted imaging (DWI) in MR without contrast has a shorter scanning time but lower spatial resolution, making it difficult to identify small lesions. However, we can observe the lesion under higher-field strength T2-weighted MR images (T2).

Interpolation-based methods (e.g., nearest-neighbor, bilinear; Kim et al., 2010; Tam et al., 2010) are simple and rapid, but they blur sharp edges and fine details (Yang et al., 2014). Recent studies have shown promising results using learning-based methods for synthesizing MR images at high-field strengths, such as sparse learning (Zhang et al., 2012) and random forests (Alexander et al., 2014). For example, Xiang et al. (2018) proposed a deep embedding convolutional neural network (DECNN) to synthesize CT images from T1-weighted MR images, Qu et al. (2020) introduced a deep learning network that leverages wavelet domain to synthesize 7 T MRI from 3 T MRI. Isola et al. (2017) proposed that conditional generative adversarial networks (GANs) not only learn image-to-image mappings, but also learn a loss function for training the mappings. GANs tend to synthesize higher-quality images while DWI MRI and T2 MRI differ in both resolution and contrast (Andrew et al., 2019).

**FIGURE 1:**
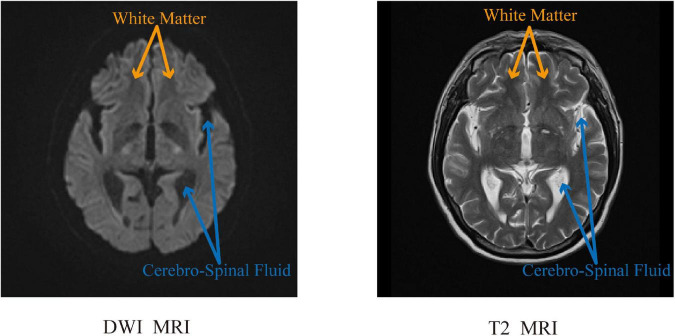
DWI MRI and T2 MRI.

**FIGURE 2:**
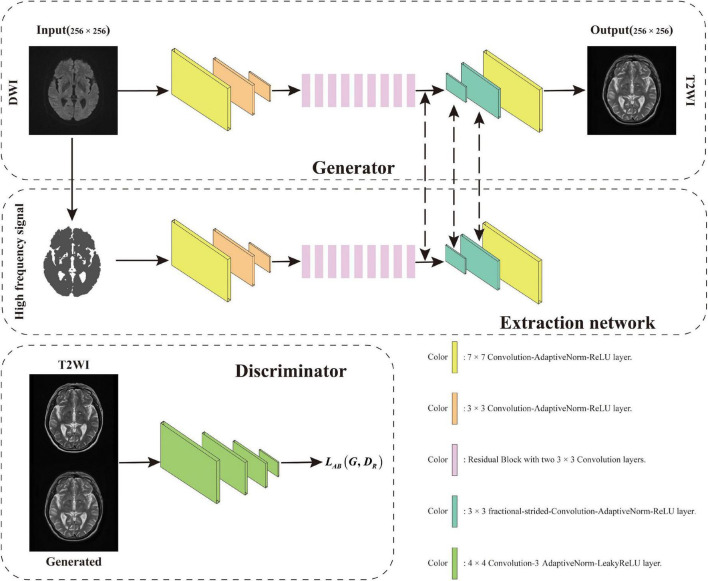
AN-GAN framework design consists of three parts, namely, generator, extraction network, and discriminator.

This article aims to address the problem of synthesizing T2 MRI from DWI MRI. When scanning a patient’s cranial MRI, the brain tissue signals collected under different pulse sequences are quite different. The large variability in these acquired brain tissue signals makes this synthesis problem challenging to solve. Recently, convolutional neural network (CNN) has become a common method for image prediction; furthermore, many studies are constantly improving this model (Liao et al., 2013; Xu et al., 2016; Huang et al., 2021). Briefly, for a given objective, CNN can automatically learn to minimize the loss function. If it takes a simple approach and asks the CNN to minimize Euclidean distance between predicted and ground truth pixels, it will produce muzzy results (Pathak et al., 2016; Zhang et al., 2016). Therefore, it would be highly desirable if we can instead specify only a high-level goal, such as “make the output indistinguishable from ground truth,” to synthesize realistic images and then automatically learn a loss function that satisfies this goal, which is also the research direction of GANs (Goodfellow et al., 2014; Denton et al., 2015). In this work, examples of the DWI image and their corresponding T2 image are shown in Figure 1. These images were taken from a cerebral MRI of the same patients. In the DWI MR image, the blue arrows point to the “cerebrospinal fluid (CSF)” with a low-intensity value, and the yellow arrows point to the white matter with a high-intensity value. However, in the T2 MR image, the “CSF” appears to be bright, while the “white matter” appears to be dark. In general, the mapping between these two sequences of DWI and T2 is highly complex. Inspired by this, we incorporate some prior knowledge, such as the difference of signals between different tissues in MRI, to guide the synthesis of the generator and make the generator become more powerful. Therefore, as shown in the generator design method of GANs in Figure 2, we introduce high-level semantic information for splicing with features extracted from the source domain and receive the input of this information in the middle layer to generate T2 MRI with richer details.

**FIGURE 3:**
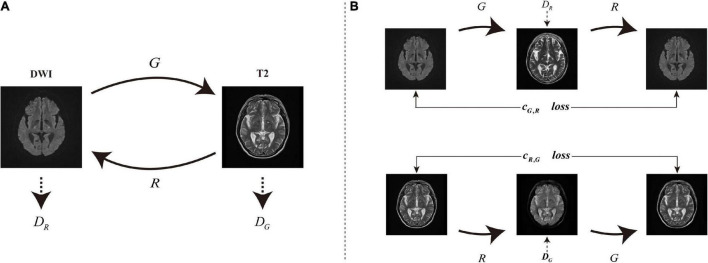
**(A)** Two generator mapping models *G*:*DWI*→*T*2 and *R*:*T*2→*DWI*, and two associated adversarial discriminators D_*G*_ and D_*R*_. D_*G*_ distinguishes between the images generated by G and T2, and vice versa for D_*R*_ and R. **(B)** Two consistency loss *c*_*G*,*R*_:*DWI*→*G*(*DWI*)→*R*(*G*(*DWI*))≈*DWI* and *c*_*R*,*G*_:*T2*→*R*(*T2*)→*G*(*R*(*T2*))→*T2* to further regularize the mappings.

In this article, we introduce GANs to solve the synthesis problem of DWI MRI to T2 MRI and use an adaptive normalization (AN) before activation function to make it suitable for image synthesis after adding high-level semantic information. Similar to the batch normalization (Ioffe and Szegedy, 2015), the activation is normalized in a channel-wise manner and then modulated with learned scale and shift. As shown in Figure 3, there are two generators *G*:*DWI*→*T*2 and *R*:*T*2→*DWI*, and associated adversarial discriminators *D*_*G*_ and *D*_*R*_. The forward generator *G* learns the mapping from DWI (source domain) to T2 MRI (target domain), and the reverse generator *R* learns the mapping from T2 to DWI MRI. We add a cycle consistency loss function (Zhou et al., 2016) that encourage the two cycle-generated behaviors *G*(*R*(*T*2))≈*T*2 and *R*(*G*(*DWI*))≈*DWI*. Finally, through the experiments we find that the images synthesized by the AN-GAN framework after using the AN method and adding high-level semantic information are effective for medical image synthesis. Some classical image synthesis methods such as pix2pix and cycle-GAN achieved better perceptual appearance; however, there could be excessive deformation in the generated images, and this may affect their clinical applications. However, the images generated by our proposed AN-GAN framework have more details.

## Related work

Recent studies on image synthesis based on learning include deep generative models. Many experts and scholars have carried out research on conditional image synthesis in order to improve the quality of synthesized images and achieved gratifying results. They have also made innovations in normalization methods.

Recent deep generative models include GANs (Goodfellow et al., 2014) and variational autoencoder (VAE; Kingma and Welling, 2014). The network we proposed is built on GANs and adds a module to extract high-level semantic information. The traditional GANs have a generator and a discriminator where the goal of the generator is to generate realistic images so that the discriminator cannot distinguish between synthetic and real images. The adversarial losses in GANs have achieved many results on image synthesis problems, such as image generation (Denton et al., 2015), image generation (Zhu et al., 2016), and representation learning (Mathieu et al., 2016). Recent studies include person image synthesis and editing (Zhang et al., 2021), image inpainting (Liu et al., 2021), and image attribute editing (Wang et al., 2021). We refer to cycle-GAN framework proposed by Zhu et al. (2017) to train two generators and two discriminators, as shown in Figure 3, where one generator learns the mapping from the source domain to the target domain and the other generator learns the mapping from the target domain to the source domain, and the two generators and the corresponding discriminators have the same structure.

The input of many problems adopts the idea of conditional image synthesis, such as many models of text-to-image synthesis (Mescheder et al., 2018; Brock et al., 2019). Recent studies include converting the semantic layouts that construct from text to images through an image generator (Hong et al., 2018) and using a single-text condition translate image styles (Kwon and Ye, 2022). Another widely used form is image-to-image synthesis based on conditional GANs, where both input and output are images. Image-to-image translation can be traced back to the work of Hertzmann et al. (2001) on image analogies, who used non-parametric models (Efros and Leung, 1999) to create new images from a single paradigm. Recent studies have enhanced the expressiveness of the generator by providing an example style map to control the style of output image (Huang et al., 2018) and extracting information from semantic layout and scene attributes as condition variables (Karacan et al., 2016). Mirza and Osindero (2014) proposed conditional generative adversarial nets that use the given labels to generate specific images in the testing phase. The “pix2pix” framework was proposed by Isola et al. (2017), who used conditional GANs to learn image-to-image mappings. Zhu et al. (2017) proposed a cycle-GAN framework on this basis using unpaired data for training. Choi et al. (2018) proposed StarGAN that implements the transfer of multiple domains using one model. Compared with earlier non-parametric-based methods such as composing realistic pictures from simple freehand sketches annotated with text labels (Chen et al., 2009), learning-based methods are generally faster during testing. In this article, DWI images and high-level semantic information extracted from DWI images are used as training sets, and the proposed AN method updates affine parameters to synthesize more detailed T2 MR images.

The normalization layer is an important part of the deep learning network now, including unconditional normalization and conditional normalization, which can be found in various classifiers. Currently popular unconditional normalization layers include instance normalization (Ulyanov et al., 2016), layer normalization (Ba et al., 2016), group normalization (Wu and He, 2018), and weight normalization (Salimans and Kingma, 2016). Conditional normalization includes conditional batch normalization (Dumoulin et al., 2017) and adaptive instance normalization (Huang and Belongie, 2017). Different from the earlier normalization techniques, conditional normalization layers require external data and generally operate as follows. First, layer activations are normalized to zero mean and unit deviation. Then the normalized activations are denormalized by modulating the activation using a learned affine transformation whose parameters are inferred from external data. In the style transfer task, these affine parameters are used to control the global style, and the spatial coordinates are consistent. However, our proposed AN applies spatially varying affine transformations and is suitable for synthesizing medical images by generators that incorporate high-level semantic information of target domain images.

## Adaptive image synthesis

The goal of this article is to learn the mapping from set *A* to set *B*, that is, the mapping function from DWI to T2 MR. Training samples {*x*_1_,*x*_2_,…,*x*_*N*_},*x*_*i*_ ∈ *A*, {*y*_1_,*y*_2_,…,*y*_*N*_},*y*_*i*_ ∈ *B*, *where N* means the number of training samples. We simultaneously train two generators *G*:*A*→*B* and *R*:*B*→*A*, and two corresponding discriminators *D*_*G*_ and _*DR*_. This training procedure is shown in Figure 3. The discriminator *D*_*G*_ distinguishes between the image {*y*} of the target domain and the image {*G*(*x*)} generated by the source domain, while the discriminator *D*_*R*_ distinguishes the image {*x*} of the source domain and the image {*R*(*y*)} generated by the target domain. Our training objective consists of two aspects, namely, adversarial loss function and consistency loss function. The former uses the data distribution of the images generated in the source domain to match the data distribution of the target domain images, and the latter prevents conflicts between the learned generators *G* and *R*. We also use the extracted high-level semantic information as a condition to guide the generator to synthesize images and use an AN method to make it suitable for image synthesis after stitching high-level semantic information.

Here, we give the design principle of cycle-GAN. We assume that there is some potential relationship between the domains. For example, they are the presentation of two different signals of brain tissues and organs. We can use supervision at the sets of level (there is one set of images in domain *A* and a different set in domain *B*) in the absence of paired data. Therefore, we design two mappings, i.e., *G*:*A*→*B* and *R*:*B*→*A*. Meanwhile, *G* and *R* should be inverse to each other, and both mappings are bijections. The goal of the mapping *G* is that the output $\hat{y}=G\,\left(x\right),x\in A$, is indistinguishable from images *y* ∈ *B*. The goal of the mapping *R* is that the output $\hat{x}=R\,\left(y\right),y\in B$, is indistinguishable from images *x* ∈ *A*. This article realizes this assumption by training the mappings *G* and *R* simultaneously. Then the consistency loss is introduced to enforce guarantee *G*(*R*(*B*))≈*B*. Finally, this loss is combined with the adversarial loss in domain *A* and domain *B* to achieve the goal of image-to-image conversion.

### Adversarial loss

The mapping functions of both generators use adversarial losses, first proposed by Goodfellow et al. (2014). Let *y* be the adversarial loss function composed of the generator *G*:*A*→*B* and the corresponding discriminator *D*_*G*_ as follows:


(1) $$L_{{\mathrm{GD}}_{G}}\,\left(G,D_{G},A,B\right)=E_{x∼P_{x}}\,\left[\log\left(1-D_{G}\,\left(\hat{y}\right)\right)\right]+E_{y∼P_{y}}\,\left[{\mathrm{logD}}_{G}\,\left(y\right)\right]$$

where $\hat{y}=G\,\left(x\right)$, *P*_*x*_ and *P*_*y*_ are the distributions of the source domain and the ground truth image. The goal of the generator *G* is to synthesize images $\hat{y}$ that look similar to the real image *y*, which is in the set *B*, while the goal of the discriminator *D*_*G*_ is to distinguish $\hat{y}$ from *y*. Similarly, the adversarial loss function composed by the generator *R*:*B*→*A* and the corresponding discriminator *D*_*R*_ can be written as follows:


(2) $$L_{{\mathrm{RD}}_{R}}\,\left(R,D_{R},B,A\right)=E_{y∼P_{y}}\,\left[\log\left(1-D_{R}\,\left(\hat{x}\right)\right)\right]+E_{x∼P_{x}}\,\left[{\mathrm{logD}}_{R}\,\left(x\right)\right]$$

where $\hat{x}=R\,\left(y\right)$. The goal of the generator *R* is to synthesize images $\hat{x}$ that look similar to the real image *x*, which is in the set *B*, while the goal of the discriminator *D*_*G*_ is to distinguish $\hat{x}$ from *x*. Finally, our goal is to make the image synthesized by the generator closer to the real image, against the discriminator that distinguishes the generated image from the real image, which can be expressed as follows:


(3) $$\min_{G}\max_{D_{G}}L_{{\mathrm{GD}}_{G}}\,\left(G,D_{G},A,B\right),\min_{R}\max_{D_{R}}L_{{\mathrm{RD}}_{R}}\left(R,D_{R},B,A\right)$$

### Consistency loss

Our model uses *l*_1_ regularization as a pixel-level constraint to penalize network in order to avoid the blurring effect of the generated images when using *l*_2_ regularization. Our discriminator adopts the structure of “70 × 70” PatchGAN. This discriminator divides image into *N*×*N* patches equally, penalizes structure at the scale of patches, and then classifies the true and false of each patch., As shown in Figure 3, we think the mapping function is cycle-consistent which can reduce unnecessary mapping between set A and set B. Therefore, we use a consistency loss to motivate the two behaviors $x\to \hat{y}\to R\,\left(\hat{y}\right)\approx x$ and $\,y\to \hat{x}\to G\,\left(\hat{x}\right)\approx y$ as follows


(4) Ladv⁢(G,R)=Ey∼Py⁢[G⁢(x^)-y 1]+Ex∼Px⁢[R⁢(y^)-x 1]

where∣∣*∣∣_; 1_ means _*l*1_ -*norm*. Our total loss function is written as follows:


(5) $$L=L_{{\mathrm{GD}}_{G}}\,\left(G,D_{G},A,B\right)+L_{{\mathrm{RD}}_{R}}\,\left(R,D_{R},B,A\right)+\lambda \,L_{\mathrm{adv}}\,\left(G,R\right)$$

where λ = 10.

### Adaptive normalization

We propose a new conditional normalization method to address the problem regarding the fusion of introduced high-level semantic information. Let *m* ∈ *Q^H×W^* be a mask, where *H* is the image height and *W* is the image width. The high-level semantic information extracted from the residual network is fused to the source domain information. Then they are characterized on *m*. First, let ^*li*^ be the activations for a batch of *N* samples in the *i*th layer in a deep convolutional network. Let ^*Ci*^ be the number of channels in this layer. Let ^*Hi*^ and ^*Wi*^ be the height and width of the activation map in this layer. Similar to the batch normalization, the pixel values after the convolution operation are normalized in a channel-wise manner, then modulate with the learned scaling parameter γ and shift parameter μ. The value in space (*n* ∈ *N*,*c* ∈ ^*Ci*^,*a* ∈ ^*Hi*^,*b* ∈ ^*Wi*^) can be expressed as follows:


(6) $$\gamma_{c,a,b}^{i}\,\left(m\right)\,\frac{h_{n,c,a,b}^{i}-\mu_{c}^{i}}{\sigma_{c}^{i}}+\beta_{c,a,b}^{i}\,\left(m\right)$$

(7) $$\mu_{c}^{i}=\frac{1}{{\mathrm{NH}}^{i}\,W^{i}}\,\sum_{n,a,b}h_{n,c,a,b}^{i},$$

$$\sigma_{c}^{i}=\sqrt{\frac{1}{{\mathrm{NH}}^{i}\,W^{i}}\,\sum_{n,a,b}\left({\left(h_{n,c,a,b}^{i}\right)}^{2}-{\left(\mu_{c}^{i}\right)}^{2}\right)}$$

where $h_{n,c,a,b}^{i}$ is the activation before normalization.$\mu_{c}^{i}$ and $\,\sigma_{c}^{i}$ are the mean and standard deviation of the activations in channel c. $\gamma_{c,a,b}^{i}\,\left(m\right)$ and $\beta_{c,a,b}^{i}\,\left(m\right)$ are affine parameters learned in the normalization layer, which depend on the high-level semantic information and vary with respect to the location (*a*,*b*). The value of mask *m* in the activation map at the site (*n*,*a*,*b*) is updated by using the scaling parameter $\gamma_{c,a,b}^{i}\,\left(m\right)$ and the translation parameter $\beta_{c,a,b}^{i}\,\left(m\right)$.

## Experiments

**FIGURE 4:**
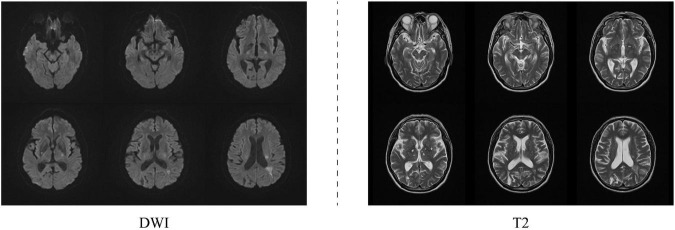
DWI and T2 datasets.

To verify the effectiveness of proposed AN-GAN, we evaluate our method through experiments on MR image translation, including descriptions of experimental data, experimental settings, and evaluation metrics. In the ablation experiments, the improvements we explore benefit from two points in the proposed framework, namely, high-level semantic information extracted from the source domain and the proposed new normalization method. To verify the effectiveness of these two modules, we will remove the high-level semantic information module in the AN-GAN framework, use a batch normalization method, and compare these two experimental results with the proposed method. Finally, we will compare with existing GANs models CGAN, pix2pix, cycle-GAN, and StarGAN. The experimental data included two MRI sequences, namely, DWI and T2. Figure 4 shows an example of these data.

### Datasets

The study was approved by the Institutional Review Board of Yantai Yuhuangding Hospital and the Ethics Committee of Shandong Technology and Business University, and informed consent from the patients was waived. The experimental data used in this article are collected from 20 adult volunteers at Yantai Yuhuangding Hospital. They were scanned under a DISCOVERY MR750w MRI scanner, using a self-shielding gradient set with a maximum gradient amplitude of 40m^T^m^−1^. The size of each DWI MR image is 256 × 256 × 16, the size of each T2 MR image is 256 × 256 × 12, and the voxel size is 1 mm×1 mm × 1 mm.

### Experimental settings

We conduct all experiments using Windows 11 with NVIDIA RTX3060 GPU and 12th Gen Intel(R) Core (TM) i5-12400F CPU, and the environment is Python3.7 and PyTorch1.8.0. Based on the scanning direction of the 3D medical images, we slice the 3D medical images scanned by all MRI equipment into 2D images and use the 2D slices to train our proposed model. When conducting experiments, we crop the size of the input image to 256×256, where the parameter λ of Eq. 5 is set to 10, the initial learning rate is set to 0.0001, and the batch size is set to 2.

**FIGURE 5:**
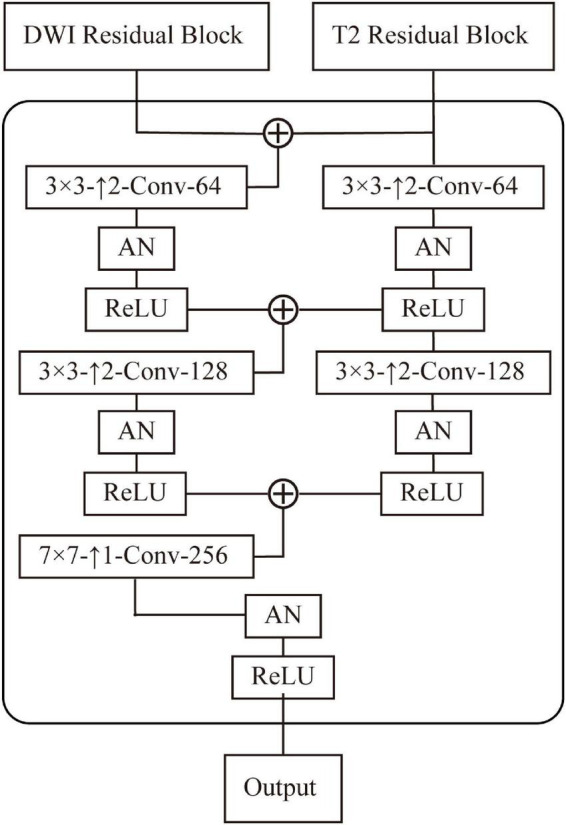
Design of the decoder of generator, which contains three deconvolution layers, where 3×3−*Conv*−64 means a 3×3 convolutional layer with 64 filters. ↑2 means a deconvolution with stride 2 operate.

The AN-GAN framework is shown in Figure 5. We normalize each layer of the network using an AN approach. The discriminator uses 70×70 PatchGANs (Isola et al., 2017). It uses fewer parameters than the full-image discriminator and can process arbitrary images in a convolutional fashion. During the training process, the adversarial loss uses a least-squares loss (Mao et al., 2017). In the process of deconvolution, the high-level semantic information of the source domain is used to guide the synthesis of the generator, where the decoder design is shown in Figure 5.

In this article, we use mean squared error (MSE), peak signal to noise ratio (PSNE), structural similarity (SSIM) (Wang et al., 2004), and feature similarity index measure (FSIM) (Zhang et al., 2011) for an objective evaluation of image translation results. The real images of all target domains are used as reference datasets, and the SSIM and FSIM scores of the generated images are used as quantitative evaluation criteria.

### Ablation study

**FIGURE 6:**
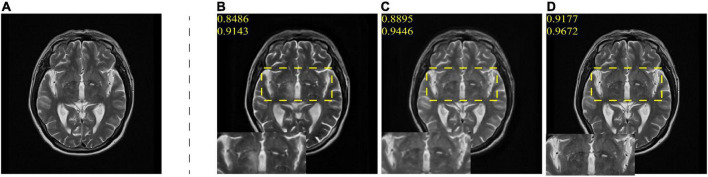
**(A)** is the real T2 image; **(B)** is the T2 image generated with mask information; **(C)** is the T2 image generated with AN; and **(D)** is the T2 images generated with mask information and AN. The numbers in yellow represent the SSIM and FSIM scores of the generated images.

To verify the effectiveness of adding AN methods and high-level semantic information, we compare AN-GAN with AN with AN-GAN using only batch normalization and AN-GAN adding only high-frequency information GANs are compared and quantitatively analyzed in our dataset. As shown in Table 1, AN-GAN using AN and high-level semantic information effectively improves image translation performance. The synthesized image is shown in Figure 6. From the CSF in the yellow box area in Figures 6B,D, it can be seen that only adding high-frequency information does not improve the quality of image translation, and the synthesized result is blurred. Among them, (Figure 6C) compared with (Figure 6D), after using high-frequency information and AN, the image structure synthesized by AN-GAN is more complete, the details are richer, and it is closer to the real image. SSIM and FSIIM scores are used for quantitative evaluation. We find that using high-frequency information and AN, SSIM achieves 0.9177 and FSIM achieves 0.9762, which are higher than the scores of 0.8486 and 0.9143 for adding high-frequency information alone and 0.8895 and 0.9446 for using AN alone.

**TABLE 1:** Quantitative assessment of MRI image conversion.

Method	MSE	PSNR	SSIM	FSIM
AN-GAN (Without AN)	111.22 ± 5	27.67 ± 1	0.85 ± 0.2	0.91 ± 0.2
AN-GAN (Without mask)	96.86 ± 5	28.27 ± 1	0.89 ± 0.2	0.94 ± 0.2
AN-GAN	86.40 ± 5	29.30 ± 1	0.92 ± 0.1	0.97 ± 0.2

### Comparison

We evaluate the feasibility and effectiveness of the AN-GAN framework that will be compared with classic models in the field of image translation as follows:

• CGAN: a method to generate a specific image. The proposal of CGAN enables GAN to use images and corresponding labels for training, and use the given labels to generate specific images in the testing phase.
• Pix2pix: a method using patch-level discriminators.
• Cycle-GAN: a method to learn two mappings simultaneously.
• StarGAN: this method implements the transfer of multiple domains using one model.

**FIGURE 7:**
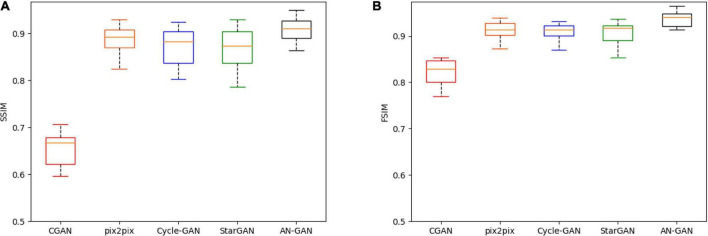
Boxplots of SSIM **(A)** and FSIM **(B)** scores for synthetic images of three network models (CGAN, pix2pix, Cycle-GAN, StarGAN, and AN-GAN) on the test set.

**FIGURE 8:**
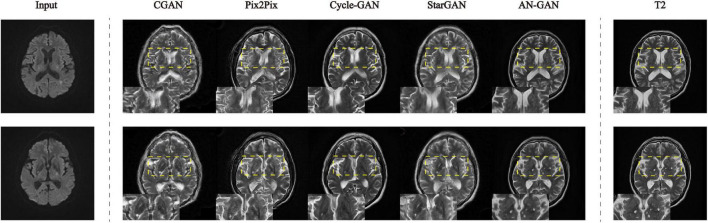
Experimental results of CGAN, pix2pix, Cycle-GAN, StarGAN, and AN-GAN in the test set. Synthesis of images from DWI to T2.

In this article, these five models are quantitatively evaluated on the test set. The results of SSIM and FSIM are shown in the boxplot in Figure 7. Compared with CGAN, pix2pix, Cycle-GAN, and StarGAN, the AN-GAN framework is more stable and the effect is better. As shown in Figure 8, the images generated by the CGAN framework have large deformations. Pix2pix, Cycle-GAN, and StarGAN frameworks all successfully implement image translation between the two domains with good results, but some brain tissues are not visually clear. The results of our proposed AN-GAN show clear details and distinct texture of soft tissue, which is superior to other methods.

## Conclusion

In various clinical scenarios, medical images are crucial for the diagnosis and treatment of diseases. Different sequences of MRI images provide doctors with different lesion information, which complement each other and help doctors make accurate decisions in clinical scenarios. However, the cost of MRI equipment is high. Using low-cost scanned MRI image sequences to synthesize other sequence MRI images can not only save the cost of patients but also provide doctors with more comprehensive lesion information. In contrast to the existing methods, this article proposes the AN-GAN framework, which adds high-frequency information to guide generator training and designs an AN method to make it suitable for generators incorporating the high-frequency information. Through experiments on our collected datasets, we demonstrate that AN-GAN outperforms other state-of-the-art methods.

## Data availability statement

The raw data supporting the conclusions of this article will be made available by the authors, without undue reservation.

## Ethics statement

The studies involving human participants were reviewed and approved by the Institutional Review Board of Yantai Yuhuangding Hospital and Ethics Committee of Shandong Technology and Business University. Written informed consent for participation was not required for this study in accordance with the national legislation and the institutional requirements.

## Author contributions

YM: conceptualization, methodology, and writing—review and editing. CC: conceptualization, software, writing—original draft, methodology, formal analysis, investigation, and validation. ZW: writing—review, editing, and validation. DC, PY, XH, BZ, and FZ: writing—review and editing. All authors contributed to the article and approved the submitted version.
